# Advances in the Application of Electrospun Drug-Loaded Nanofibers in the Treatment of Oral Ulcers

**DOI:** 10.3390/biom12091254

**Published:** 2022-09-07

**Authors:** Yangqi Zhou, Menglong Wang, Chao Yan, Hui Liu, Deng-Guang Yu

**Affiliations:** 1 School of Materials Science and Engineering, University of Shanghai for Science and Technology, Shanghai 200093, China; 2 Shanghai Engineering Technology Research Center for High-Performance Medical Device Materials, Shanghai 200093, China

**Keywords:** electrospinning, mucoadhesive polymers, drug delivery, nanofiber film, oral ulcers

## Abstract

Oral ulcers affect oral and systemic health and have high prevalence in the population. There are significant individual differences in the etiology and extent of the disease among patients. In the treatment of oral ulcers, nanofiber films can control the drug-release rate and enable long-term local administration. Compared to other drug-delivery methods, nanofiber films avoid the disadvantages of frequent administration and certain side effects. Electrospinning is a simple and effective method for preparing nanofiber films. Currently, electrospinning technology has made significant breakthroughs in energy-saving and large-scale production. This paper summarizes the polymers that enable oral mucosal adhesion and the active pharmaceutical ingredients used for oral ulcers. Moreover, the therapeutic effects of currently available electrospun nanofiber films on oral ulcers in animal experiments and clinical trials are investigated. In addition, solvent casting and cross-linking methods can be used in conjunction with electrospinning techniques. Based on the literature, more administration systems with different polymers and loading components can be inspired. These administration systems are expected to have synergistic effects and achieve better therapeutic effects. This not only provides new possibilities for drug-loaded nanofibers but also brings new hope for the treatment of oral ulcers.

## 1. Introduction

The main functions of the mouth include chewing, sucking, swallowing, speech, feeling, expression, ingestion of food, participation in breathing, etc. The mouth is an indispensable organ for people in their daily life [1]. Oral ulcers are a highly prevalent mouth disease that affects both men and women, young and old. They not only cause pain to patients but also interfere with normal physiological activities, such as eating and speaking. Oral health is identified by the World Health Organization as one of the top ten standards of human health [2]. Mouth diseases not only affect the physiological function of the mouth but also have different degrees of impact on the health of the whole body. Recurrent oral ulcers can directly affect the body’s immune function, bring about various complications, and even cause cancer. The causes of oral ulcers are complex and varied. There are significant individual differences between patients in terms of the cause and extent of the disease. Therefore, there is no uniform and mature treatment method. At present, there are common forms of oral ulcer treatment on the market, such as gargle, cream, gel, bulk, and tablet. These dosage forms are frequently administered and tend to mix with saliva in the mouth. In contrast, the oral patch is more suitable to apply in the treatment of mouth ulcers. It has the advantage of adhering to the ulcer site, thus delivering the drug locally and protecting the ulcer wound [3,4,5,6,7]. Thus, it prolongs the action of the drug, reduces the number of doses, and alleviates pain. In addition, nanofibers can be loaded with multiple drugs and control drug-release rate. This promises a synergistic effect between drugs for quickly and effectively healing. This oral nanofiber patch can be simply prepared by high-voltage electrospinning.

High-voltage electrospinning is a single-step technique for preparing nanofibers. Electrospinning enables a direct transition from liquid to solid nanofibers. Nanofibers and nanoparticles have great potential for applications in various fields, such as medicine [8,9], tissue engineering [10,11,12], sensors [13,14,15], environment [16,17], energy [18], self-healing polymers [19], and food [20]. Electrospinning is extremely flexible in terms of material selection, drug loading, and structural design, making it particularly suitable for medical applications.

Many polymers can be electrospun. In addition, a lot of drugs, functional particles, proteins, and peptides can be successfully loaded into nanofibers. The different combinations of these polymers and loading components have given rise to infinite possibilities. In terms of structure, nanofibers with different three-dimensional structures can be produced simply by changing the spinnerets with different structures (coaxial, Janus, triaxial, etc). These structures often allow for different drug-releasing behaviors [21,22,23,24]. For example, coaxial or Janus nanofibers obtain a biphasic release of drugs through polymers with different hydrophilic properties [25]; hydrophilic polymers can be prepared by triaxial electrospinning to achieve sustained release of core–shell nanofibers [26]; triaxial nanofibers achieve zero-order controlled release of drug [27]. In conclusion, nanofiber film has outstanding advantages in the field of drug delivery.

**Figure 1:**
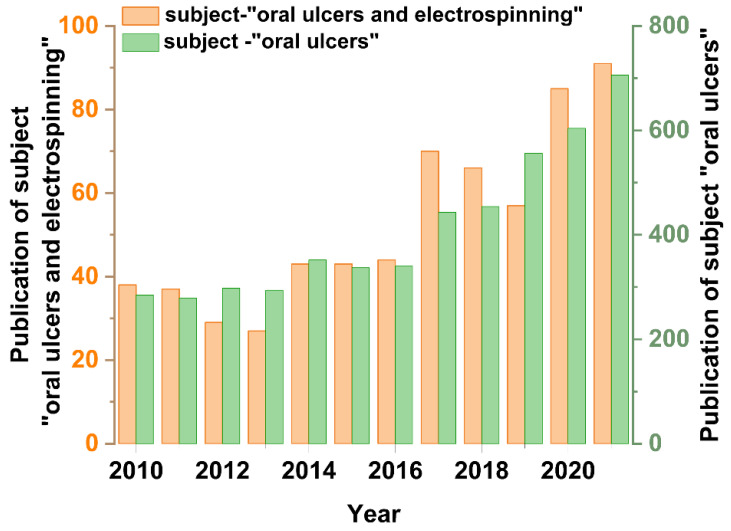
Number of publications on “oral ulcers” and “electrospinning technology for oral ulcers” since 2010.

Nanofiber films have many applications studied, such as a wound dressing on the skin surface [28], whereas the mouth environment is very different from the skin. The wetting and flexibility of the oral mucosa places additional demands on the nanofiber film. Nanofiber film has to adhere to the ulcer site and release the drug. Therefore, polymers applied in the treatment of mouth ulcers have good biocompatibility and bioadhesive properties. For the loading component of the nanofiber, first, it must be able to be stably dispersed in the polymer solution. Secondly, it must be able to achieve pain relief and antibacterial or anti-inflammatory functions to reduce symptoms or shorten healing time. Studies have been conducted to treat mouth ulcers using nanofibrous films to aid in ulcer healing [29,30,31,32]. Therefore, nanofiber film is feasible and advantageous in the treatment of mouth ulcers. However, few studies have been conducted so far about the application of nanofiber films prepared by electrospinning technology to treat mouth ulcers. A search of Web of Science showed the number of publications on the topics of “oral ulcers” and “oral ulcers and electrospinning” for each year since 2010 (Figure 1). This showed that the number of publications in both areas is increasing each year. Surprisingly, the number of publications on “electrospinning and oral ulcers” represents almost one-tenth of all studies on “oral ulcers.” However, with the development of electrospinning technology, the application of nanofiber membranes for the treatment of mouth ulcers is receiving more and more attention. It has been documented that drug-loaded nanofibers are effective in accelerating the healing time and extent of oral ulcers, but there is no uniform pattern among studies. Summarizing the polymers and specific ingredients used in the various protocols will help to arrive at better combinations to achieve better therapeutic results. Electrospinning can also be combined with other techniques (solvent casting, etc.) to treat mouth ulcer diseases. This gives new ideas and hopes for the application of electrospinning. In conclusion, it is meaningful to study the application of electrospinning technology in the treatment of oral ulcers.

## 2. Oral Ulcers

**Figure 2:**
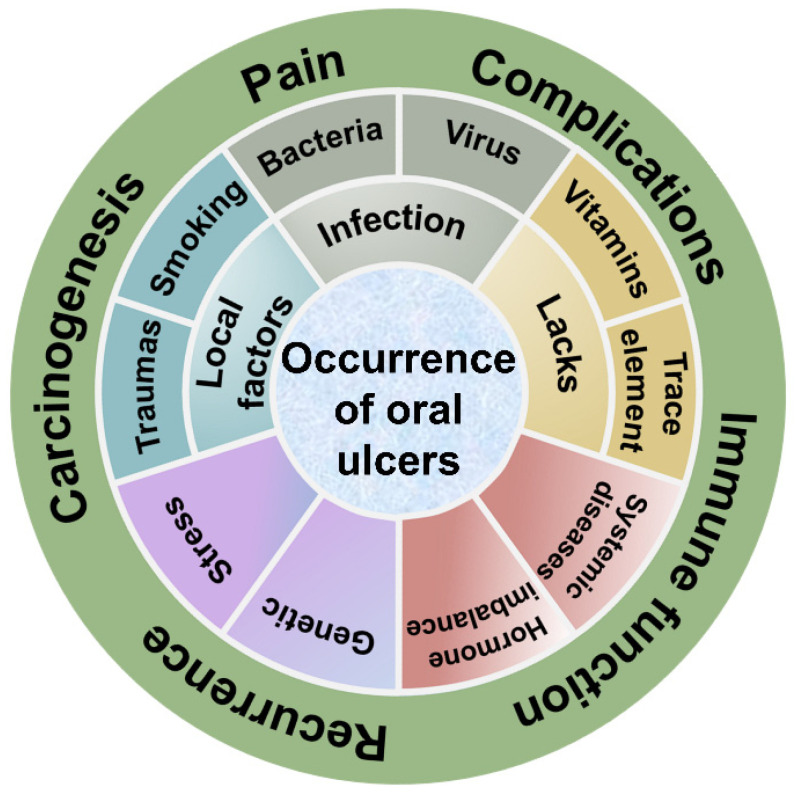
The causes and dangers of oral ulcers.

The incidence of oral ulcers is as high as 5–20% and one of the most prevalent oral diseases [33]. Patients are both men and women, young and old, with young and middle-aged people being the most numerous. The trauma of oral ulcers is mostly found in areas where the mucosa lacks a keratinized layer or is poorly keratinized (inner lip, tongue belly, buccal mucosa, vestibular groove, and soft palate) [34]. The exact cause of mouth ulcers remains elusive (Figure 2). Currently, clinical treatment of oral ulcers is still focused on promoting local wound healing.

As a recurring oral mucosal disease, oral ulcers have many dangers [33]. First, when the ulcer attacks and the pain is severe and brings great inconvenience to daily life. Secondly, when the oral ulcer is recurrent, it will directly affect the immune function of the whole organism of the patient [35]. Systemic complications include halitosis, chronic pharyngitis, constipation, fever, headache, dizziness, swollen lymph nodes, and loss of vision. Thirdly, if a painful oral ulcer is accompanied by ulcers in other parts of the body, high priority should be given to them. This is because such ulcers are likely to be a warning sign of immune disease. Fourth, long-term untreated oral ulcers might point to the possibility of cancer. If the ulcer is more than 1 cm in diameter with elevated edges and a depressed center, the depressed surface has granular bumps. If hard lumps can be felt around and at the bottom of the ulcer, it may be a sign of cancer [36].

### 2.1. Oral Mucosal Structure

**Figure 3:**
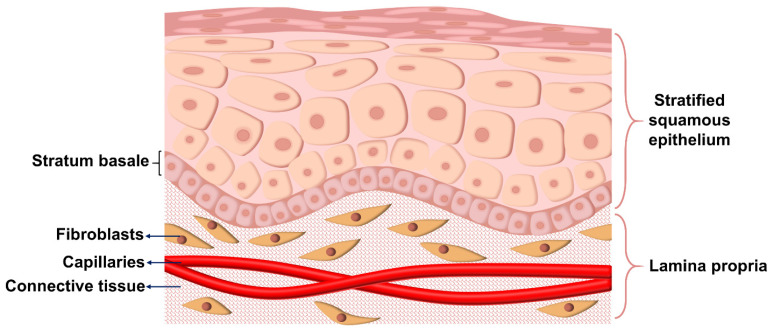
Schematic diagram of the organization of the oral mucosa.

Most oral ulcers occur in the oral mucosa. The oral mucosa is all the wet tissue covering the inner surface of the mouth from the red lips (the red lips are the migration between the skin and the mucosa) inwards. The oral mucosa is divided into the epithelium and lamina propria (Figure 3) [37]. The mucosal epithelium is composed of several layers of cuboidal cells. The further away from the basal lamina, the flatter and more elongated the nucleus of the cells [38]. Human oral keratinocytes comprise a stratified squamous epithelium with keratinization only in the hard palate. The lamina propria is tightly connected to the epithelial pegs by protrusions. Fibroblasts produce fibers and extracellular matrix to form the lamina propria. The superficial layer of the lamina propria consists of loose connective tissue, blood vessels, and nerve tissue. The deeper layer of the lamina propria consists of dense connective tissue and a lot of fibers. The rich capillaries within the lamina propria give the mucosa its red color.

In different regions of the oral cavity, the cells of the oral mucosal epithelium are differentiated between keratinocytes and non-keratinocytes. The keratinized epithelium is distributed on the gingival and dorsal surface of the tongue. The keratinized squamous epithelium is mainly divided into four layers: basal layer, echinoderm layer, granular layer, and stratum corneum. The basal layer is the innermost layer of the epithelium. Basal cells are formed by the division of stem cells. In addition, a portion of stem cells will divide and differentiate into epithelial cells to maintain the dynamic balance of proliferation and shedding of mucosa cells. The echinoderm layer is located outside the basal layer and developed from the proliferating basal cells. The protrusions between the cytoplasm are connected as bridge grains, and the cells are connected by bridge grains. The substances of the bridge granules are proteins, of which transmembrane proteins play an important role in the adhesion. The granular layer is located outside the echinoderm and is made up of two to three levels of fat cells. Keratinized cells are the outermost layer and consist of keratinized or incompletely keratinized flat cells. Similarly, the non-keratinized epithelium is divided into four main layers: basal layer, echinoderm layer, mesoderm layer, and superficial layer. However, the non-keratinized epithelium does not have the superficial layer of keratinization. The non-keratinized oral mucosal epithelium is distributed with a lot of cells that are not involved in epithelial cell proliferation and maturation. These cells include melanocytes, Langerhans cells, and Merkel cells. Among them, Langerhans cells are antigen-presenting cells, which are closely related to the immune function of the mucosa. From a functional and histological point of view, there are three main categories of oral mucosa. The first category is the lining mucosa, which includes the buccal mucosa, lip mucosa, and so on. This category has the largest range of mucosa and belongs to the non-keratinized stratified squamous epithelium [39]. The second category is the masticatory mucosa, which is found on the dorsum of the tongue and gingival. The epithelium of this category of mucosa is keratinized. The third type of mucosa in the area of taste perception on the dorsal surface of the tongue and the lingual papillae.

The histological structure of the oral mucosa is similar to that of the skin, but there are differences. First, most mucous does not have a cuticle. Second, the basal cells of the mucosa are rectangular in shape, whereas the basal cells of the skin are cylindrical. Third, although the epithelial cells of both evolve from the basal cells, the hierarchy and morphology between the epithelial cells of the mucosa are less regular than that of the skin. Fourth, there are more blood vessels in the lamina propria of the mucosa. Therefore, the oral mucosa has a higher permeability than the skin to facilitate drug absorption.

### 2.2. Drug Absorption in the Oral Mucosa

The thickness, blood supply, and degree of keratinization of the oral mucosa vary in different locations within the oral cavity. As a result, the rate and extent of drug absorption vary from region to region. The oral mucosa is a lipid structure, and the main route of drug absorption through the oral mucosa is passive diffusion. A few substances, such as D-glucose, amino acids, and vitamins, are transported and facilitated by active diffusion in the oral cavity. The epithelium is the primary drug-delivery target for the treatment of most oral mucosal diseases [38]. Oral ulcer trauma is mostly seen in the nonkeratinized layer of the mucosa or areas with poorly keratinized. Nonkeratinized epithelial sites are more easily permeable to drugs for systemic absorption.

The degree of keratinization is inversely proportional to the permeability of the mucosa [37]. The order of drug permeability from strong to weak is sublingual mucosa, buccal mucosa, gingiva, and hard-palate mucosa. In addition, the physicochemical properties of the drug are also important factors affecting the degree of oral mucosal drug delivery. The larger the molecular mass of the drug, the more difficult it is to pass through the oral mucosa. When the molecular mass of the drug is higher than 800 Da, it is difficult to pass through the oral mucosa [40]. The lipid and aqueous solubility of the drug affects the way it is absorbed into the oral mucosa. If the lipid solubility of the drug is too strong, it is difficult for the drug to reach the effective level in saliva. If the lipid solubility is too weak, it cannot be absorbed through the lipid barrier. In addition, oral mucosal administration requires drugs to be stable in the pH range of 5.5–7.0 [41]. Besides local administration, the oral mucosa can also be used for systemic administration. The approach for common oral ulcers is a local treatment, which is the most effective way to improve ulcer symptoms. The drug is applied directly to the lesion of the mucosa and works at the site of application. For frequently recurring or long-term untreated oral ulcers, systemic administration is used [42]. The drug is absorbed from the oral mucosa and directly enters the body’s circulation, avoiding the first-pass effect on the liver. This avoids the destruction of the drug by pH value and enzyme system of the gastrointestinal tract. The oral mucosal adherent membrane enables local and systemic treatment, making it an ideal modality for the treatment of oral ulcers.

### 2.3. Causes and Types of Oral Ulcers

The causes of oral ulcers are complex. The causes may be local trauma (physical injury, chemical injury); vitamin or trace element deficiency; bacterial or viral infection; genetic factors; systemic diseases (peptic diseases, gastric ulcer, diarrhea and constipation, lupus erythematosus); hormonal imbalance; allergies; psychospiritual factors (stress); or lifestyle factors (smoking) [43]. In addition, oral ulcers are also common after chemotherapy or radiotherapy. Local trauma is an important etiology of oral ulcers, where trauma leads to epithelial edema and early inflammatory cell infiltration of the oral mucosa [44]. Common activities where trauma damages the oral mucosa are chewing too hot and hard foods, improper gargling, biting the lips, etc. Certain micronutrient deficiencies can also cause oral ulcers, such as the micronutrients zinc, iron, folic acid, and vitamin B. In addition, microorganisms may play an important role in the occurrence and development of oral ulcers. There are approximately 700 species of bacteria colonizing the human oral cavity, and bacteria can act as pathogens to produce a direct inflammatory response. Streptococcus and Helicobacter pylori are involved in the development and progression of oral ulcers. Studies on the monogenic inheritance and polygenic inheritance of oral ulcers have shown that there is a genetic predisposition to oral ulcers. If parents have recurrent oral ulcers, 90% of their children have oral ulcers. If parents have no oral ulcers, only 20% of their children have oral ulcers [33]. Hormonal imbalances can also affect oral ulcers. It has been observed that women’s oral ulcers are more likely to worsen during hormonal fluctuations such as menstruation, luteal phase, and menopause. Psychological factors can affect the immune system. Studies have shown that people who are stressed, anxious, and depressed are more likely to suffer from oral ulcers than those who are less stressed. Smoking can aggravate the pain of oral ulcers and prolong the healing time, and can even lead to oral tissue lesions that can cause oral cancer.

**Figure 4:**
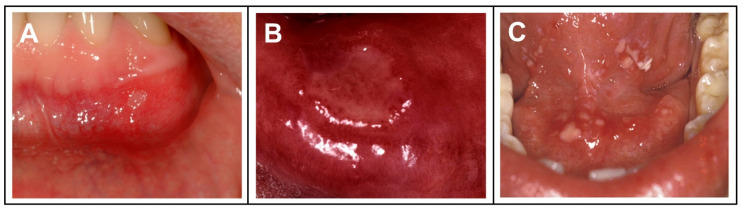
Schematic diagram of a patient with oral ulcers. (**A**) Mild oral ulcer, reprint permission from Ref. [45]. Copyright 2008, Churchill Livingstone; (**B**) severe oral ulcer, reprint permission from Ref. [45]. Copyright 2008, Churchill Livingstone; (**C**) herpes-type oral ulcers, reprint permission from Ref. [46]. Copyright 2020, Association for Dental Sciences of the Republic of China.

Different etiologies bring about different clinical manifestations. Usually, recurrent oral ulcers are classified into three categories: mild, heavy, and herpetic (Figure 4). The symptoms, morphology, number, distribution site, and duration of disease differ among the three types of oral ulcers and are summarized in Table 1 [33]. The process of oral ulcer healing mainly includes injury response, inflammatory response, and cell proliferation and differentiation. Cell death occurs during oral mucosal injury and inflammatory cytokines are activated. Subsequently, epithelial cells migrate, proliferate and differentiate into a new extracellular matrix, and finally, the ulcer heals.

### 2.4. The Ideal Dosage Form for the Treatment of Oral Ulcers

At present, the common dosage forms on the market for the treatment of oral ulcers are mouthwash, cream, gel, film, and so on. Mouthwash is used frequently and the active ingredient has a short residence time, so it tends to have only some antibacterial effect. Creams and gels are easily flushed by saliva, and the drugs do not stay effective. The films are thinner, used less frequently, and have good therapeutic effects to protect ulcerated wounds. In short, the film is the most suitable dosage form. High-voltage electrospinning allows simple and flexible preparation of nanofiber films. The high surface area and porosity of nanofiber films make them thin and flexible. Certain polymeric substrates rapidly absorb water and swell, and interact with the mucus of mucosa. Thus, they stay in the treatment site for a long time, avoiding and disadvantages of other dosage forms that are frequently used.

#### Advantages of Electrospun Nanofibers in Drug Delivery

Nanofiber films that can adhere to ulcer wounds are ideal dosage forms for the treatment of oral ulcers, with the following advantages. (1) Electrospinning is simple to prepare and the results are reproducible. Moreover, nanofiber films can be prepared in various sizes and shapes for easy use. (2) Nanofiber films can be designed in multiple layers. The hydrophilic and hydrophobic layers cooperate to achieve unidirectional drug release and improve drug utilization. (3) The nanofiber film is in continuous close contact with the mucosa and adheres to the wound site for a long time. This facilitates improved drug absorption and reduces the number of drug administration. (4) Prevents the first-pass effect of the drug and degradation of the active ingredient by enzymes or acids in the gastrointestinal tract. After absorption through the oral mucosa, the drug enters the body cycle directly through the lid vein, internal maxillary vein, and brachial vein. This not only avoids gastrointestinal and hepatic first-pass effects but also improves the bioavailability of the drug. Just as oral ketoprofen (KET) may cause gastrointestinal side effects, mucosal administration is a safe alternative to oral administration [29]. (5) Nanofiber films can be administered locally or systemically to reduce pain and accelerate ulcer healing. (6) The drug is easily absorbed. The oral mucosa does not have a cuticle barrier like the skin. Therefore, the permeability is better than the skin, and the drug is easily absorbed through the oral mucosa. (7) The nanofiber film is soft and thin, which improves patient compliance. It is convenient to administer medication and can be used anywhere, anytime, and without water. It shows certain advantages in medication administration for children. (8) Nanofiber film not only improves physical protection against secondary wound injury.

## 3. High-Voltage Electrospinning

### 3.1. Principle of High-Voltage Electrospinning

High-voltage electrospinning is a simple and practical new technology capable of generating nanostructures directly from large-scale spinnerets. The electrospinning process is accomplished through the interaction between hydrodynamics, electrodynamics, and rheology. The electrospinning solution is propelled through the pump to form small droplets at the tip of the needle. The charged polymer droplets overcome surface tension by electric field forces to form a jet of fine flow. The liquid jet is bent, stretched, and split under the combined effect of electrostatic repulsive forces, Coulomb forces, and surface tension. Meanwhile, rapid evaporation of the solvent and solidification of fine streams lead to the direct formation of nanofiber aggregates, which are collected into nanofiber films.

**Figure 5:**
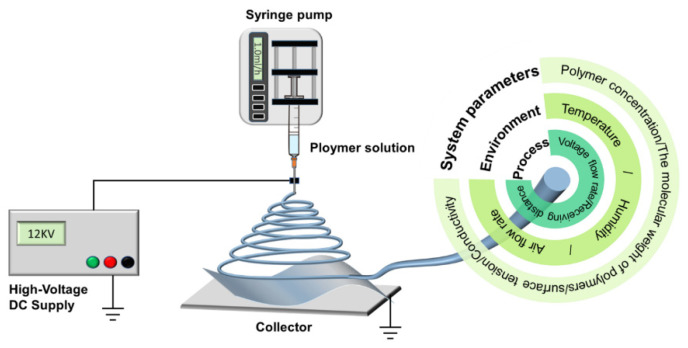
Schematic diagram of high-voltage electrospinning and the factors influencing the diameter of nanofibers.

The basic equipment of electrospinning is divided into four main parts (Figure 5). One is the high-voltage DC power supply, which provides the power to make the solution ejected. Adjusting the waveform and frequency of the high-voltage alternating current can improve the spinnability and productivity of nanofibers [47]. The second is the fluid-driving device, which is the core part of electrospinning and is used to control the flow rate of the solution. The third is the spinneret. The spinneret is connected to the fluid drive. The biggest advantage of electrospinning is that nanofibers with corresponding structural characteristics can be produced simply by designing and modifying the spinneret structure. This advantage is not available with various other “bottom-up” chemical synthesis methods. Fourth is the receiving device. The receiving device is usually wrapped in a layer of aluminum foil outside the plate and connected to the ground. Receiving distance is also an important parameter of the electrospinning process.

Nanofiber diameter is closely related to properties. When nanomaterials process the scale of matter to the nanoscale, their physical or chemical properties change compared to those at larger scales. These new properties make it superior in drug delivery. Nanofiber films have a structure similar to that of the extracellular matrix. The finer the nanofibers, the higher the specific area and the higher the pore size, thus providing more active sites and better drug-release properties. Nanoscale porous materials are superior in the field of antimicrobial materials [48]. In addition to porosity, grooved nanofibers also bring such value as helping nerve regeneration [49].

Nanofiber diameter is mainly related to three parameters: environment, process, and system (Figure 5) [50]. The environmental parameters include temperature and humidity. Within a certain range, the diameter of nanofibers decreases at higher temperatures. When the humidity is too high, water droplets condense on the fiber surface, making it porous after drying. The higher the air circulation ratio, the faster the evaporation of the solvent, so the more porous and larger diameter of the nanofiber may be. The process parameters include voltage, flow rate, and receiving distance. As the voltage increases, the nanofiber diameter first decreases and then increases. Within a certain range, the stronger the jet stretching with higher voltage, the smaller the diameter of the nanofibers. However, as the voltage increases further, the flow rate of the spinneret increases and the nanofiber diameter increases. In matching the voltage, the larger the stream rate, the higher diameter, but too large a stream rate can produce a string bead structure. The larger the receiving distance, the longer the stretching time and the nanofiber diameter decreases. The system parameters are mainly related to the polymer properties. The polymer has to reach a certain concentration before it can be electrospun. When the concentration is too low, the viscosity and chain entanglement of the polymer solution is too low. As such, the polymer solution cannot be ejected and only droplets can be collected. High concentration of the polymer leads to larger nanofiber diameter. This is because the viscosity increases as the intermolecular Coulomb forces strengthen with increasing polymer concentration. The larger the molecular weight of the polymer, the thicker the nanofiber. The higher the surface tension, the longer the jet time and the smaller the nanofiber diameter. The diameter of nanofibers decreases with increasing electrical conductivity. However, the bending and whipping processes are unstable, leading to a broadening of the diameter distribution.

Electrospun drug-laden nanofibers can improve drug-encapsulation rate and drug-site contact area. In addition, nanofiber films have a unique structure similar to the extracellular matrix and good biocompatibility, providing sites for cell attachment, growth, and proliferation. Electrospinning allows a flexible choice of polymers and loading components. Many polymers are biodegradable and biocompatible. Some of these polymers (e.g., poly(α-L-glutamic acid)) are also nonimmunogenic and suitable as drug-delivery matrices [51]. Combining different water-soluble polymers with drugs can achieve rapid release of insoluble drugs or slow release of active ingredients. Various structures of spinnerets correspond to the nanofibers of that structure to achieve different drug-release rates. In conclusion, the potential of electrospinning technology is constantly explored and has developed rapidly in recent years.

### 3.2. Development of High-Voltage Electrospinning

Initially, electrospinning could only convert polymers into nanofibers. However, this mere transformation was of little value, and the development of electrospinning experienced a long period of stagnation afterward. It was not until the later discovery that small-molecule drugs, active ingredients, and nanoparticles could be easily encapsulated into nanofibers that the significance of electrospinning was greatly expanded. Nanomaterials have been greatly developed for medical, environmental, food, and other applications. In the medical field, a nanofiber-integrated negative sorting device can be used for cancer diagnosis [52]. Nanocomposites with shape-memory properties enable the fabrication of shape-programmable bone scaffolds [53]. Core–shell lipid-coated nanoparticles and nanodelivery systems of melittin can be used for cancer therapy [54,55]. Biodegradable nanoparticles hold promise for diabetes treatment [56]. Keratin/poly(butylene succinate) nanofibers may aid in wound healing [57]. Nanoparticles deliver central nervous system drugs for migraine [58], and nanofibers deliver antimicrobial drugs for endodontic disease [8]. Mucosal adhesion microspheres deliver nifedipine for the treatment of hypertension [59], and coaxial nanofibers transdermally deliver varenicline tartrate for smoking cessation [60]. In the environmental field, electrospinning can prepare MOFs for wastewater treatment applications. Metal–organic framework (MOF) composites have excellent adsorption properties for the removal of heavy metal ions and organic dyes, among others [61]. Functional nanomaterials prepared by a combination of calcination and electrospinning can degrade antibiotics in water [62]. Polyacrylonitrile (PAN) nanofibers embedded with MgO can adsorb copper ions from copper-containing wastewater and recycle them [63]. Hybrid materials made of polymeric nanofibers and photocatalytic nanoparticles can be applied to polluted water remediation [64]. In addition, the nanofiber membrane is one of the suitable air-filter materials [65,66]. A Ta-MOF–polyether block amide nanohybrid can adsorb the gaseous pollutant CH_4_ [67]. In the food sector, preparing traditional seasonings such as soy sauce into nanocomposites can improve their portability [20]. Chitosan (CS) nanofiber composite films containing *Urtica dioica* leaf extract can extend the shelf life of packaged guava fruit [68].

Nanofibers prepared by electrospinning technology have significant advantages over conventional materials, especially for drug-delivery systems in the medical field. The preparation of drug-loaded nanofibers by electrospinning was first reported in a conference report in 1993 [69]. Since then, there has been a rapid development in the structure, properties and applications of electrospinning. Nanofibrous membranes can support cell adhesion and angiogenesis [70,71]. Biocompatible poly(amino acid) nanofiber membranes can achieve effective hemostasis and repair of the wound [72]. Nanofiber mats prepared from natural polymers and alkannin and shikonin (A/S) derivatives not only facilitate cell attachment and proliferation but also provide high antimicrobial activity [73]. It is worth mentioning that A/S derivatives promote wound healing and provide antibacterial and analgesic effects on ulcerated wounds [70]. Loading of A/S derivatives into nanofibers is ideal for topical and transdermal drug delivery [74]. Thus, when cellulose acetate was used as a matrix, nanofiber films loaded with A/S derivatives showed outstanding antimicrobial properties [71]. Nanofibers can be used as dressings for wounds resulting from many different causes, including foot ulcers due to diabetes [75]. Nanofiber mats containing cinnamon essential oil and nanoparticles are effective for chronic diabetic wound healing [76]. In addition, nanofibers have great potential as wound sutures [77]. In addition to regular and uniform-sized nanofibers and particles, beaded nanofibers have been found to have great potential for drug encapsulation. Beads can act as a repository for drugs, reducing release at the initial stage to achieve slow drug release [78]. Electrospinning technology continues to be explored for specific medical applications.

In addition to solid components, nanofiber film releases nitric oxide (NO) to help with wound contraction, antibacterial action, and angiogenesis. However, small-molecule NO donors are not stable by themselves and cannot achieve sustained release. S-nitrosated keratin (KSNO) can be used as NO donor to solve this problem [79]. KSNO was co-electrospinning with poly(ε-caprolactone) to obtain small-diameter vascular grafts. It was experimentally shown to inhibit thrombosis and act as rapid endothelialization and vascular remodeling in vascular tissue engineering [80]. Polypropylene (PU)–KSNO nanofiber film was successfully prepared by electrospinning by Dou et al. The nanofiber film was shown to release NO continuously for 72 h to aid wound healing [79]. KSNO mixed with PU and gelatin (Gel) to prepare nanofiber films was shown to have proliferation-promoting effects on L929 murine fibroblasts and human umbilical vein endothelial cells [81].

**Figure 6:**
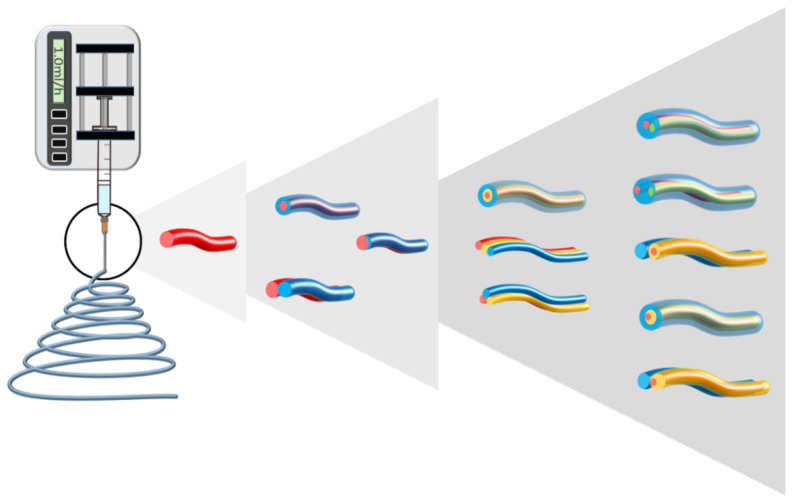
Structural development of high-voltage electrospinning.

The rapid progress of electrospinning technology is largely reflected in the change in nanofiber structure. For a long time, electrospinning could only produce uniform single nanofiber. Generations of studies followed for the next hundred years. Finally, in 2003, Sun et al. accomplished a breakthrough in the history of electrospinning by producing nanofibers with a core–shell structure [82]. This breakthrough inspired a lot of research scholars, and research advances on the variations of electrospinning structures follow in droves. As the number of fluids increases, the style of the structure rises exponentially (Figure 6). Multifluid electrospinning greatly expands the ability of electrospinning. The non-electrospinnable working fluid can work with the electrospinnable fluid, making the electrospinning process smooth. Two-fluid electrospinning has Janus, coaxial, and other structures that help the nanofibers achieve more precise controlled drug release. Three-fluid electrospinning even brings an unexpected linear drug-release rate. In addition, nanofibers can obtain hollow structures, etc. The relationship between structure and performance has been further explored to solve numerous drug-delivery challenges.

**Figure 7:**
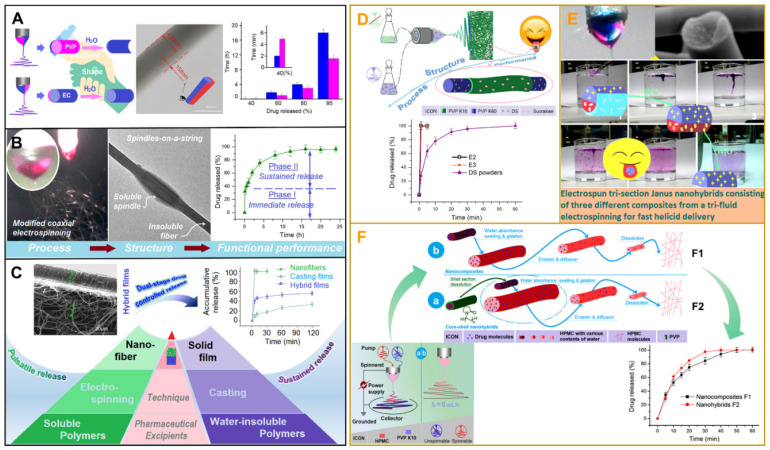
Performance triggered by transformation of electrospinning structures. (**A**) Improved coaxial electrospinning for biphasic drug release, reprint permission from ref. [21]. Copyright 2021, Elsevier; (**B**) String beaded nanofibers for biphasic drug release, reprint permission from Ref. [22]. Copyright 2021, Springernature; (**C**) The combination of cast film–nanofiber membrane for biphasic drug release [84]; (**D**) Improvement of coaxial (solvent circulation) to improve the solubility of the insoluble drug [23]; (**E**) Trisection Janus nanofibers for rapid release of insoluble herbal medicine, reprint permission from Ref. [24]. Copyright 2022, Springernature; (**F**) The core–shell nanohybrids for rapid release of the insoluble drug [87].

Changes in the structure of nanofibers have led to radical advances in performance, especially in the field of drug delivery. Biphasic drug release has practical significance [83]. Biphasic drug release can be achieved by different methods (Figure 7A–C). Biphasic release refers to a rapid release of an effective drug concentration followed by a slow sustained release. This is to maintain an effective drug concentration for a while. Biphasic drug release can be achieved by using two different water-soluble polymers. Polyvinylpyrrolidone (PVP) achieves rapid release of ibuprofen (IBU) in the initial phase, while hydroxypropyl methylcellulose (HPMC) achieves sustained release of IBU. This combination achieves a biphasic release of the drug, which facilitates pain relief and reduces gastric irritation. The controlled release of IBU with high stability and low irritation has been achieved [25]. The improved Janus structure achieves a more precise biphasic release of the drug compared to the regular Janus structure. Using different water-soluble polymers (hydrophilic PVP and hydrophobic ethyl cellulose (EC)), the crescent-shaped surface provides better rapid release and the circular surface provides better-sustained release (Figure 7A) [21]. Nanofibers with beads achieved an apparent biphasic release with two different phases of release (Figure 7B) [22]. In addition, the combination of a slow-releasing cast film with a fast-releasing nanofiber film also allows the drug to achieve biphasic release (Figure 7C) [84].

The problem of delivery and absorption of insoluble drugs, a key issue in drug-delivery systems, can be effectively addressed by nanoscale fibers. IBU is a typical drug with poor water solubility. To improve its dissolution properties, its presence in a highly dispersed amorphous state in fibers is an ideal solution. Ning et al. developed a new oral dispersion membrane using the refractory drug diclofenac sodium (DS) as a model to achieve rapid release of the refractory drug. In addition, a pure solution can be used as a sheath fluid in electrospinning. It makes the preparation process smooth and improves the smoothness of the nanofiber surface (Figure 7D) [23]. Oral dispersion films can be a good way to improve patient compliance [85]. A novel trisection Janus nanofiber was prepared to deliver helicide, a poorly water-soluble herbal medicine. By using different polymers in the three parts, a rapid and sequential release of helicide was achieved. This offers the possibility of drug delivery through the tongue mucosa (Figure 7E) [24]. Chinese herbal medicine (Lianhua Qingwen Keli) used for the treatment of the novel coronavirus pneumonia can be rapidly released by electrospun hybrid films [86]. In addition, the structure of nanofibers affects the solubility of insoluble drugs. The core–shell nanohybrids were more capable of enhancing acetaminophen (AAP) release compared to single-fluid electrospinning of HPMC and AAP composites (Figure 7F). The modified coaxial electrospinning uses HPMC-AAP as the core and PVP and sucralose composites as the shell. This improves nanofiber water absorption and HPMC gelation, resulting in the rapid release of AAP [87].

**Figure 8:**
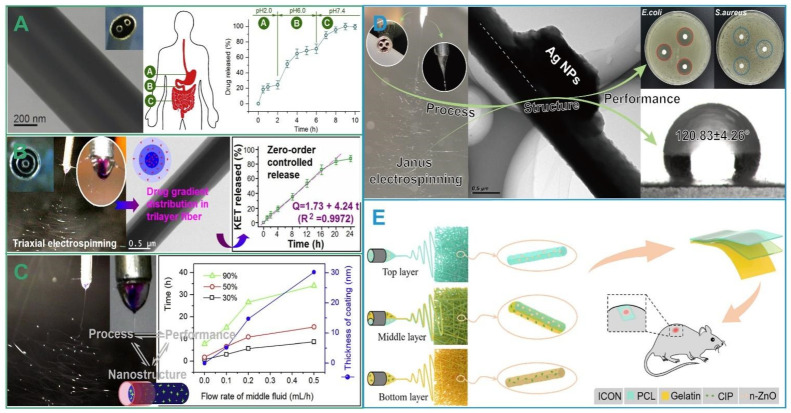
Significance of specially structured nanofibers. (**A**)The sheath-separate-core structures for targeted drug delivery [88]; (**B**) Triple-layer nanofibers for zero-order drug release, reprint permission from Ref. [90]. Copyright 2015, American Chemical Society; (**C**) Triaxial electrospinning for zero-order controlled release, reprint permission from Ref. [27]. Copyright 2019, Elsevier; (**D**) Nanofibers with synergistic antibacterial effect for wound dressing [92]; (**E**) Multilayer nanofibers as wound dressing, reprint permission from Ref. [93]. Copyright 2022, Elsevier.

Special structure enables the need for targeted drug delivery. The sheath-separate-core structure of the nanofibers uses Eudragit S (ES) 100, a polymer with pH-dependent solubility and spinnability. ES 100 can target drugs to the colon (Figure 8A) [88]. ES 100 is not dissolved in the acidic environment of the stomach and releases the drug in the neutral colonic site. Functionalized selenium nanoparticles can be used as a carrier for liver-targeted drug delivery [89]. When ordinary drugs enter the body, only a very small fraction can act on the lesion and have toxic side effects. Targeted drug delivery can reduce toxic side effects and drug waste and improve therapeutic efficacy.

Zero-order release is the release of a drug that maintains a constant rate during the release cycle. The complex structure of multifluid electrospinning can successfully achieve this release mechanism. A triple-layer nanofiber with an increasing drug-concentration gradient was successfully prepared by triaxial electrospinning. This triple-layer nanofiber achieved a zero-order release of the model drug (KET) within 20 h and has practical applications (Figure 8B) [90]. Improvements to electrospinning can further control drug release. Core–shell nanofibers are prepared by electrospinning using an electrospinnable solution as the core fluid and a non-electrospinnable liquid as the external and intermediate working fluid. This core–shell nanofiber can not only achieve zero-order release over a while but can also regulate the release rate by sheath layer thickness (Figure 8C) [27]. By controlling the flow rate of the sheath solution, the drug-loading and drug-release profile can be adjusted [91]. Subtle changes in structure have led to countless changes in performance. The correspondence between structure and performance has triggered continuous innovation by countless researchers.

The application of electrospinning technology to wound dressings is developing rapidly. The loading components in the nanofibers can be natural oils and metal particles in addition to drugs. The Janus nanofibers exerted a synergistic antibacterial effect on lavender oil and silver nanoparticles (Figure 8D) [92]. Nanofiber films can also be collected into multiple layers to better promote wound healing using the cooperation of different layers (Figure 8E) [93]. The composite of hydrogels and nanofibers allows the delivery of drugs with two different release profiles. The composites are useful in soft-tissue tumor treatment [94].

**Figure 9:**
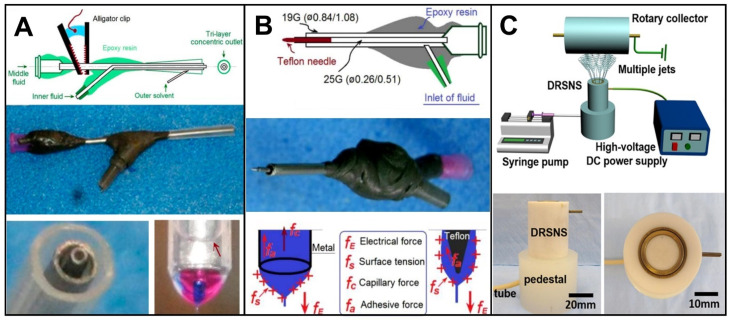
Progress of electrospinning in energy saving and high yield. (**A**) Energy-saving spinneret Figure [96]; (**B**) Energy-saving solid Teflon-core rod with core spinneret [97]; (**C**) Highly productive DRSNS, reprint permission from Ref. [30]. Copyright 2019, American Chemical Society.

In addition to achieving structural transformation, nanofiber quality also continues to advance. In 2010, Yu et al. broke the concept of “shell layer must be electrospinnable.” Improved electrospinning with solvent circulation can improve the continuity of the electrospinning process and the quality of nanofibers [95]. The use of polyglycolic acid (PP) tubes as the outermost layer of the spinneret can also improve the quality of the nanofibers (Figure 9A). In addition, using PP tubes as the outermost layer of the spinneret has an energy-saving effect. It prevents electrostatic energy from escaping into the environment [96].

Energy saving is of great importance in the development of electrospinning. To reduce cost, a solid Teflon-core rod is inserted into the core of the common coaxial spinneret (Figure 9B). This modified spinneret is similar to the nanofibers produced by conventional spinnerets. Similarities include morphology, dimensional distributions, in vitro dissolution rates, and in vitro drug-penetration rate. Conventional single-fluid electrospinning requires a high voltage of 9.4 KV and a current of 0.06 mA to sustain the stable operation, whereas, the solid core electrospinning only requires an applied voltage of 7.6 KV and a response current of 0.04 mA for stable operation. Experiments have proven that this new spinneret consumes only 53.9% of the energy of conventional electrospinning. In principle, the Teflon rod effectively avoids the reverse capillary forces of traditional metal spinnerets. In addition, the Teflon rod has little surface adhesion, which helps Taylor cone formation. According to the schematic diagram, it can be seen that the improved spinneret not only does not have f_c_ but also has less f_a_ than the metal surface. Both of these aspects help to improve electrical efficiency [97]. Subsequently, Wang et al. coated the receiver plate with epoxy resin, which effectively prevents the diffusion of high-voltage electrostatic energy to the environment. This further saves electrical energy and reduces the cost of high-voltage electrospinning technology [98]. The application of nonconductive materials to electrospinning provides a new idea for energy saving.

The development of electrospinning is also reflected in productivity. For a long time, the default electrospinning spinneret could only eject one stream of fluid at a time with a very small throughput. Wei et al. developed a double-ring slit needleless spinneret (DRSNS) to prepare drug-loaded nanofiber films in large quantities, breaking the limitation of conventional electrospinning single-strand jets (Figure 9C) [30]. DRSNS consists of four parts: shell, core, inner ring, outer ring, with multiple jets formed in a circular narrow slit. DRSNS not only improves the productivity of nanofibers but also improves needle clogging. Comparing the PLLA nanofibers fabricated by single spinneret and DRSNS, both exhibited smooth and uniform appearance. However, the diameter and diameter distribution of the PLLA nanofibers prepared based on DRSNS were larger and had higher mechanical properties. It is feasible to increase the productivity of electrospinning technology.

So far, a lot of studies have applied nanofibers to local or implanted drug-delivery systems [99]. A variety of electrospun medical devices are being prepared for market launch for surgical grafts or tissue regeneration [38,100]. Research institutes around the world are already studying electrospinning and nanofibers.

## 4. Electrospinning in the Treatment of Oral Ulcers

### 4.1. Polymers for the Oral Cavity

Polymers are divided into two categories—natural and synthetic. Most polymers are good drug-delivery matrices [51], but only a few are available for oral applications. Furthermore, polymers applied for the treatment of oral ulcers represent only a small fraction of the polymers that can be used in the oral cavity. Polymers such as polylactic acid [101], polyglycolic acid [102], and PCL [102] have good biocompatibility and degradability. Although these polymers have been extensively studied for use in the oral cavity to guide periodontal tissue and bone regeneration [103,104]. These polymers cannot be applied to the oral mucosal alone. This is because these polymers are not hydrophilic and do not adhere to the oral mucosa.

The use of a polymeric matrix with excellent bioadhesive properties is an ideal way to provide drug delivery to the oral mucosa. The oral cavity has a unique environment that includes constant saliva secretion and flushing, a highly moist environment, digestive enzyme breakdown, and continuous oral vibration and wear. Therefore, the selection of polymers for the treatment of oral ulcers requires consideration of biocompatibility, mechanical strength, and bioadhesives. The mucosal adherent film can prolong the residence time of the drug-delivery system, control the drug-release rate and improve drug bioavailability. Therefore, it is suitable for the treatment of oral ulcers.

**Figure 10:**
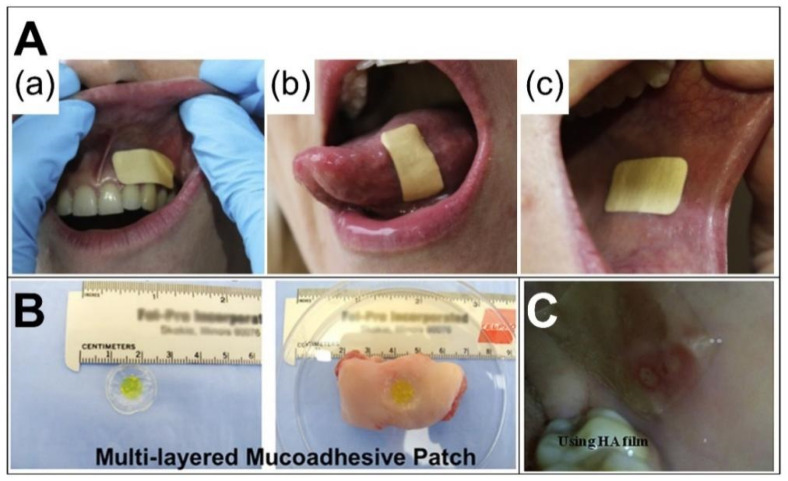
Application diagram of the oral film: (**A**) (**a**–**c**) are different applied oral places [7]; (**B**) reprint permission from Ref. [30]. Copyright 2019, American Chemical Society; (**C**) reprint permission from Ref. [108]. Copyright 2020, Elsevier.

Oral adhesion films have rapidly evolved over the last decade [105], enabling bioadhesion and long-term drug delivery (Figure 10). The Rivelin^®^ patch is one oral adhesion film that has been developed in recent years to enable long-term adhesion (Figure 10A) [7]. It was developed by Murdoch et al. at the University of Sheffield in collaboration with Dermtreat, Copenhagen, Denmark. The patch is prepared by electrospinning of adhesive polymers and is capable of treating oral lichen planus and oral canker sores [106]. The Rivelin patch is a typical layered system. The adhesive layer absorbs water to adhere and release steroids or drugs. The backing layer provides a unidirectional drug release, protecting healthy tissue from the drug. The innovation of the Rivelin patch is that it has a longer adhesion time than any current treatment method. This reduces the dose of drug required for treatment and allows the drug to enter the diseased tissue directly. Typically, an oral adhesion film consists of two layers (backing layer and adhesion layer) or a single layer (adhesion layer). It allows for two types of drug delivery. One type is the unidirectional drug release to the oral mucosa. The other type is a bidirectional drug release to the mucosa and the oral cavity. The backing layer is made of an insoluble polymer and the adherent layer is made of a dissolvable adherent polymer. The adhesive layer not only serves as a drug carrier but also as a link between the drug-carrying layer and the nonadhesive layer. The bioadhesive buccal patch is the most suitable drug-delivery system for oral mucosa [107].

### 4.2. Bioadhesive Polymers for the Oral Cavity

Bioadhesion is the tight adhesion between the adhesive material and the mucus or epithelial cells. The presence of large amounts of saliva in the oral cavity connects the mucoadhesive material to the mucosa. Saliva is a hydrophilic, viscous fluid in which the main component, mucin, gives the mucus its gelation and adhesion properties. The good wetting conditions of the oral mucosal surface bring the swellable polymer material into close contact with it. Then, the molecular chain segments of the polymer are embedded in the cellular interstices or interpenetrated with the viscous chain segments in the mucus. Finally, the polymer is tightly bound to the mucosa through a combination of mechanical embedding, covalent bonding, electrostatic attraction, hydrogen bonding, etc. The strength of adhesion is related to the polymer material properties.

Different polymers have different adhesion mechanisms, which are related to surface tension, dehydration, electrostatic interactions, and chemisorption. There are five main adhesion mechanisms: wetting theory, diffusion theory, electronic theory, fracture theory, and adsorption theory. The strength of adhesion is related to the molecular weight, wettability, swelling rate, charge density, molecular space configuration, solubility, and concentration of the polymer. Polymers have large relative molecular masses and are dispersive, and polymers have different types of molecular shapes: linear, branched, and cross-linked. The adhesion of polymers is low at high molecular weights. Polymers with a linear molecular shape spread more readily into bio-tissues and therefore have better adhesion. The adhesion is from low to high for cationic polymers, neutral polymers, and anionic polymers. Carboxyl polymers have the strongest adhesion among the anionic polymers. The wettability and swelling rate of the polymer are important factors that affect the adhesion strength and the duration of the interaction. Solvent molecules are much smaller in size and diffuse faster than the polymer. Therefore, when the polymer is in contact with a liquid, the solvent molecules can penetrate inside the polymer, causing it to expand in volume [109]. Swelling is a phenomenon unique to polymeric materials and is a prerequisite for adhesion. The hydrophilic polymer swells extremely easily in water, and the swollen polymer is in close contact with the substrate to increase the adhesion area. Then, as much as possible, it contacts and acts with mucosal proteins. The faster the polymer swells, the faster the interaction with the mucin occurs and the stronger the adhesion ability. In addition, the hydrogen bonds or ionic interactions generated between the polymer and mucin can prolong the adhesion time.

The ideal nanofiber film for the treatment of oral ulcers should have suitable adhesion and little irritation. Moreover, the nanofiber film needs to adhere stably to the oral mucosa over time, independently of salivary secretion and physiological movements of the mouth. A few polymers meet these requirements, which are summarized in Table 2.

**Table 2:** Polymers for oral mucosal adhesion.

Category	Polymer	Function	Ref.
Natural	CS	Mucoadhesive; antibacterial	[110,111,112,113,114]
	HPMC	Mucoadhesive; inhibits drug crystallization	[5,115]
	tragacanth gum (TG)	Mucoadhesive; antioxidation	[113,116,117]
	Hyaluronic acid (HA)	Mucoadhesive; anti-inflammatory	[6,118]
	SA	Mucoadhesive; hemostasis	[3,114,116,119]
	Gel	Mucoadhesive	[120,121]
Synthetic polymers	PVA	Mucoadhesive	[5,6,111,117,118,122,123]
	Sodium carboxymethylcellulose	Mucoadhesive	[120,123]
	(CMC-Na)	Mucoadhesive	[4,122,124]
	Carbopol	Mucoadhesive	[3,7,114]
	Polyethylene oxide (PEO)/ Polyethylene glycol (PEG)	Mucoadhesive	[7,117,125]

#### 4.2.1. Natural Substances for Oral Mucosa Adhesion

Some natural substances have excellent bioadhesive properties of their own, and some even have such functions as hemostasis and sterilization. Natural products are a viable way to treat wounds [126]. TG is a natural plant gum that increases the viscosity of the aqueous phase. Moreover, TG has antioxidant activity, which can accelerate wound healing [127]. Various natural substances can be used in combination. The ionic gelation method was used to prepare microspheres that could adhere to the gingival area using the polymers guar gum and TG and SA [116]. CS is a typical natural substance with bioadhesive properties. CS achieves bioadhesive properties through electrostatic interaction with negatively charged mucosal surfaces and hydrogel properties [105]. CS is a natural polysaccharide with a positively charged amine group. CS can be used as a coating material for drug carriers to control the release behavior of negatively charged molecules (trypan blue, heparin, fibroblast growth factor, epidermal growth factor, etc.). In addition, CS itself has the property of accelerating the rate of wound healing and promoting bone formation. CS has been used in wound dressings, implant scaffolds, and dental applications [128]. Cellulose acetate (CA) nanofiber with CS and propolis promotes burned skin repair [129]. Samprasit et al. electrospun CS-based nanofiber mats adhered in the oral cavity to prevent dental caries. The nanofibers exerted a synergistic antibacterial effect on garcinia mangostana (GM) extract and CS [110]. The application of natural substances as polymeric substrates is an ideal approach, but usually, natural substances are difficult to electrospin alone.

#### 4.2.2. Natural–Synthetic Polymers for Mucosal Adhesion

The combination of natural substances with synthetic polymers yields a smooth electrospinning process and good bioadhesive properties. Nanofibers prepared from TG and synthetic polymers not only provide antimicrobial properties but also aid in the adhesion and proliferation of human fibroblasts. Synthetic polymers that are often used in combination with natural substances and have adhesive properties include PVA, CMC-Na, Carbopol, PEO, PEG, PVP, etc. These synthetic polymers are water-soluble, nontoxic, and biocompatible and are widely used in various biomedical applications. CS is difficult to electrospin alone and has been combined with other polymers, such as PVA/PEO in numerous studies. Szabo et al. combined PVA/CS to prepare nanofiber films for mucosal adhesion and rapid release of antifungal agents. This improved the topical efficacy of terbinafine hydrochloride [111]. Recently, Stie et al. developed an adherent and hydrophobic bilayer membrane for sublingual drug delivery. The adherent layer consists of peptide-loaded CS and PEO. The hydrophobic layer repels saliva to aid in the unidirectional release of the drug [112]. SA is a natural anionic polysaccharide with water solubility, biocompatibility, bio-degradability, and adhesion properties. SA can be prepared into hydrogels, porous sponges, nanofibers, and other substances, which are widely used in biomedical fields. SA/PVA nanofibers can be used as wound dressing [130]. PEO is a hydrophilic synthetic polymer with excellent biocompatibility. PEO can provide nanofiber film-controlled drug-release capability and adhesion when used in electrospinning. Tort and Acarturk used electrospinning glutamine-loaded PEO/SA nanofibers for the treatment of oral mucositis in cancer patients. The nanofibers showed good mucosal adhesion, mechanical properties, stability, and release of more than 85% of the drug within 4 h [3]. Both CS and SA are biodegradable and have mucosal adhesion properties. Importantly, both polymers are soluble in water, which avoids the toxicity of other organic solvents. Therefore, these two polymers are commonly used in biomedical applications. In addition, solutions of these two natural substances have unique groups, which make them electrically charged. Cationic chitosan and anionic alginate spontaneously form ionic complexes through strong electrostatic interactions. A study has prepared coaxial nanofibers by combining natural substances (CS and SA) and synthetic polymers (PEO) by electrospinning. The coaxial nanofiber has SA/PEO as the core layer and CS/PEO as the sheath layer. This coaxial nanofiber has the potential to encapsulate the delivery of proteins, cells, and enzymes [114]. Derivatives of CS are also commonly used in the pharmaceutical field. Chen et al. prepared two types of core–shell nanofibers based on carboxymethyl chitosan (CMCS) and CMC-Na. It can adhere to the oral mucosa and deliver carvedilol (Car). The shell layers were CMCS/PVA and CMC-Na/PVA, and the core layer was a mixture of PVP, phospholipid, and Car. It was shown that both CMCS/PVA and CMC-Na/PVA could adhere to the oral mucosa and promote drug penetration [123].

Carbopol has been used worldwide in bioadhesive drug-delivery systems. As a hydrophilic polymer with excellent adhesion properties, Carbopol has a linear swelling phenomenon. Through mutual repulsion between anions, Carbopol can absorb water and swell 100 times in a short time to form a gel. When a carbomer comes in contact with saliva, the carboxyl group and mucin can rapidly produce hydrogen bonds, thus adhering to the oral mucosa [124]. Combining Carbopol and PVA to prepare the shell layer of nanoparticles greatly improves the adhesion properties of nanoparticles [122]. A nanofiber patch applied to treat periodontal injuries was fabricated using the polymers Carbopol and PAN. The nanofiber patch exhibited anti-inflammatory, mucosal adhesion, and rapid drug-release rate. This patch had good mucosal adhesion properties and required approximately 4 N/m^2^ to separate from the mucosal adhesion matrix [4].

HPMC is a common hydrophilic cellulose derivative with good bioadhesive properties. HPMC is listed by FDA and EMA as a safe delivery agent [131]. It has been widely used for 50 years as a drug matrix for the oral and oral mucosal sites. HPMC is stable in the pH range 3.0–11.0 and is enzyme-resistant [131]. A large number of hydroxyl groups are present in the HPMC molecule. These hydroxyl groups can interact with the glycoproteins in the mucus and form hydrogen bonds, resulting in adhesion. In addition, HPMC has the ideal property to prepare amorphous solid dispersions to increase the solubility of insoluble drugs [115]. HPMC with different viscosity grades and molecular weights can provide different physicochemical properties. In general, the higher the viscosity of HPMC, the faster the dissolution. After HPMC absorbs water, the pores are rapidly blocked to form a hydrogel. The hydrogel inhibits the exchange of liquid and thus serves to control drug diffusion. This hydrogel material effectively increases the time of drug release and prolongs its therapeutic effect [132]. Dott et al. combined HPMC and PVA to prepare nanofibers with good adhesion properties. Nanofibers are received onto casting membranes to form porous, fast-releasing active ingredient delivery matrices for oral mucosal drug delivery. In addition, the backing film ensures that the appropriate drug concentration is retained at the treatment site for an extended time [5].

HA has been shown to provide anti-inflammatory and mucosal adhesion [108]. HA can be used for skin tissue regeneration [133]. Since HA exhibits very high viscosity at very low polymer concentrations, HA by itself cannot be electrospun effectively. Therefore, the blending of HA and PVA can help electrospinning to proceed smoothly and prepare nanofiber membranes for application in the treatment of periodontitis. The nanofiber film not only has suitable mechanical strength but also presents a well-controlled drug-release capability [6]. The efficient and simple electrospinning technique enables the production of nanofiber patches with a high specific area and bulk ratio, as well as biodegradability. Such patches not only enable local mucosal drug delivery and prolong drug residence time but also reduce side effects.

Both natural ingredients and synthetic polymers have their advantages. To choose a polymer, first, it should be miscible with the solution and the drug. Secondly, it is important to ensure that the electrospinning process is smooth. Finally, it is important to obtain the biocompatibility, adhesion properties, and mechanical properties required for the drug-delivery system. To summarize, many studies have shown that polymers that can be used in the oral cavity have adhesion properties. First, it can provide ideas for nanofiber films for the treatment of oral ulcers. Secondly, it can lay the foundation for developing nanofiber films for this application again in the future.

#### 4.2.3. Rivelin Formulation and Clinical Trials

PVP and Eudragit RS100 are often used in combination. PVP is often used as an excipient in pharmaceutical formulations because it is not involved in human metabolism. PVP is bio-compatible and soluble and has been approved for use by the US FDA. The well-known Rivelin formulation is composed of PVP and Eudragit RS100 drug-carrying fibers and a PCL backing film. The Rivelin formulation has been patented by AFYX Therapeutics. Colley et al. prepared a mucoadhesive nanofiber film by combining PVP, Eudragit RS100, and PEO. This nanofiber film was then combined with a hydrophobic protective layer made from heat-treated PCL nanofibers to form a bilayer membrane system. This Rivelin-formulated patch adheres to the oral mucosa for topical delivery of clobetasol-17-propionic acid (Figure 10A) [7]. In human volunteers with healthy mucous membranes, the patch extended when finger-pressed. The patch showed very rapid swelling and adhesion within 60 min. The residence time was 118 min in the gums, 93 min in the oral mucosa, and 43 min in the tongue. The healthy volunteers rated the size, appearance, taste, and experience of the patches as good. In addition, Clitherow et al. prepared electrospun patches delivering lidocaine hydrochloride in the Rivelin formulation. It was investigated as a topical delivery of dental local anesthetic in the oral mucosa [134]. Clitherow et al. added medium-chain saturated fatty acids to the Rivelin formulation for the suppression of oral candidiasis [125].

A clinical trial of clobetasol patch (Rivelin-CLO) was conducted in 138 patients (99 women and 39 men, mean age 61.1 years) twice daily for 4 weeks. The patch showed an appropriate dwelling time (approximately 2 h) and good patient acceptability in the treatment of patients with oral lichen planus. Patients treated with Rivelin-CLO showed a significant reduction in ulcer size compared to placebo. The data showed that 20 μg Rivelin-CLO patch treatment demonstrated significant improvements in ulcer size (*p* = 0.047), symptom severity (*p* = 0.001), disease activity (*p* = 0.022), pain (*p* = 0.012) and quality of life (*p* = 0.003) [135]. Currently, most oral electrostatic spun patches are in the research phase. In vivo testing of electrospun patches has been primarily in animal models, and few have been tested on oral mucosa in humans. Some electrospun fiber films have shown good adhesion properties on isolated porcine mucosa though. However, the adhesion time was greatly reduced when applied to human buccal mucosa [136]. Specifically, the Rivelin patch is the only such device to date that has been tested in humans and has shown significant clinical efficacy. It is expected to be the first such electrospun mucoadhesive patch on the market [38].

### 4.3. Active Pharmaceutical Ingredients Used for Oral Ulcers

Similarly to normal wound healing, the healing process of oral ulcers consists of three main overlapping phases: the inflammatory phase, the proliferative phase, and the mature phase. However, inflammatory cells and inflammatory factors (such as IL-1α, IL-1β, TNF-α, etc.) are less frequent during the mouth ulcer recovery procedure. As a result, angiogenesis in the oral mucosa is also faster, so the inflammatory and proliferative period is shorter. Most treatments for oral ulcers are pain relief and antibacterial and anti-inflammatory. This can lessen the seriousness of mouth ulcer lesions and the danger of subsequent infection. Some treatment regimens include additional healing-promoting ingredients to shorten the healing time. Table 3 concludes the components loaded in the nanofibers used for the treatment of oral ulcers. The therapeutic results of each component on the healing of oral ulcers are analyzed. The summary could serve as a basis for inspiring more combinations. This is expected to exploit the synergy between ingredients to achieve better therapeutic effects.

**Table 3:** Ingredients used in the treatment of oral ulcers.

Materials	Polymers	Solvent	Form	Ref.
KET	Eudragit L (EL)/ES	Ethanol	Nanofiber film	[29]
Aspirin	Pullulan/CS	Aqueous acetic acid	Composite nanofiber fast- dissolving oral films	[137]
Ornidazole (OD)	HPMC/PVA/CS	Purified water; acetic acid	Solvent-cast double-layer film	[138]
Short- to medium-chain fatty acids	PVP/Eudragit RS100/PCL	DCM/DMF	Nanofiber film	[125]
GM extract	CS/Thiolated chitosan (CS-SH) /PVA	Distilled water; HCL	Nanofiber film	[110]
Terbinafine	CS/PVA	Distilled water; Polysorbate 60; glacial acetic acid	Nanofiber film	[111]
Mycotoxin	Gel	Hexafluoropropylene	Semi-interpenetrating network Gel nanofiber scaffolds	[121]
Triamcinolone acetonide	Gliadin/EC	Ethanol; Acetic acid	Nanofiber film	[139]
DEX	HPMC/PVA/CS	Purified water; acetic acid	Solvent-cast double-layer film	[138]
Ziziphus jujuba extract	PEG/PAN	DMF	Nanofiber film	[4]
Human growth hormone (hGH)	EudragitVRL100 /CS	DMAc; ethanol	Double-layered nanofiber film	[32]
Curcumin (CUR)	PLLA	Distilled water; DS	Double-layered nanofiber film	[30]
Adipose tissue- derived stem cells (ADSCs)	Collagen	Acetic acid	Soft porous freeze-dried collagen-based scaffold	[31]
Glutamine	CS/PEO	Distilled water; glacial acetic acid	Nanofiber film	[3]

#### 4.3.1. Nonsteroidal Anti-Inflammatory Drugs (NSAIDs)

Oral ulcers are usually accompanied by a burning or painful sensation that affects the patient’s daily life. NSAIDs have significant analgesic and anti-inflammatory effects. NSAIDs include aspirin, IBU, bendamine, AAP, diclofenac, paracetamol, and phentermine. These ingredients have a relatively weak analgesic effect, no addictive properties and are widely used, in addition to having a good anti-inflammatory effect. During treatment, the pain-relieving ingredient should be released quickly to immediately relieve the patient’s pain. However, NSAIDs (e.g., aspirin) are not only poorly water-soluble but also have side effects on the stomach. High-voltage electrospinning can solve such problems. Therefore, Qin et al. prepared CS/branched starch composite nanofibers to fast-dissolving oral films (FDOFs) with rapid release of aspirin by high-voltage electrospinning. FDOFs were fully soluble in aqueous within 1 min, with good thermal stability and rapid solubility [137].

**Figure 11:**
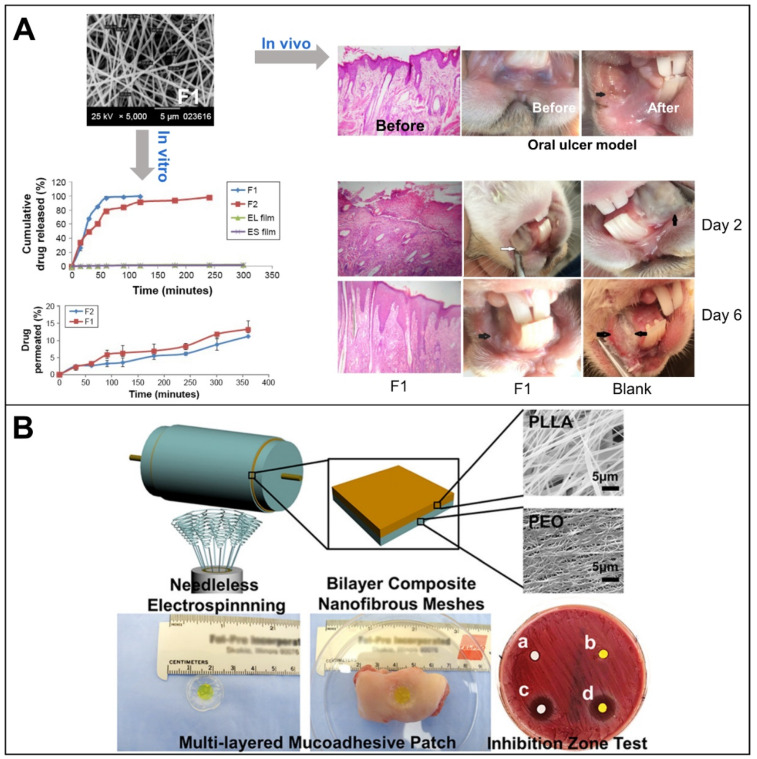
NSAIDs in the treatment of oral ulcers. (**A**) EL and ES nanofibers loaded with KET, reprint permission from Ref. [29]. Copyright 2017, Dove Medical Press Ltd.; (**B**) CUR-PLLA nanofibers loaded with DS, reprint permission from Ref. [30]. Copyright 2019, American Chemical Society.

Similarly, KET can relieve pain and suppress the inflammatory response of oral ulcers. EL and ES nanofibers loaded with KET were prepared by electrospinning (Figure 11A) [29]. It can treat oral ulcers and avoid the gastrointestinal side effects caused by oral KET administration. KET exists stable in the amorphous form in nanofibers with a 90% drug loading rate. The permeation experiments showed that the permeation rate of KET was only 11–13% after 6 h. This indicates that the local application of this nanofiber can avoid systemic absorption and side effects. Compared with EL or ES nanofibers, EL and ES nanofibers have excellent rapid-release capability, releasing more than 90% of KET within 2 h. The effectiveness of this nanofiber on oral ulcers was demonstrated by establishing an in vivo model of oral ulcers in rabbits. The nanofiber film suppressed inflammation and promoted ulcer healing. The nanofiber F1 containing KET reduced the clinical severity of oral ulcers in rabbits and healed within 6 days compared to the untreated group.

DS belongs to one of the typical representatives of NSAIDs. Wei et al. prepared multilayer nanofiber films on a large scale by a modified high-voltage electrospinning technique with a DRSNS (Figure 11B). First, PLLA nanofiber nets loaded with CUR were prepared by electrospinning. Then, PEO nanofibers loaded with DS were prepared by continuing electrospinning directly on the PLLA nanofiber nets. Finally, the bilayers were combined with HPMC adhesive films to form multilayer patches. It was found that CUR alone did not exhibit any antimicrobial effect. However, better antimicrobial activity was obtained when combining DS and CUR than when DS was used alone. This solution has simultaneous anti-inflammatory, antioxidant, antibacterial and analgesic effects, providing a new idea for the treatment of oral ulcers [30]. The double-layer PVA patch prepared by combining electrospinning technology and the freeze–thaw process can realize transdermal delivery of DS [140]. In conclusion, NSAIDs loaded into nanofibers proved to be effective in the healing of oral ulcers.

#### 4.3.2. Antifungal Agent

The ulcer site has reduced defenses against bacteria and bacterial colonization of the wound site can cause infection. Infection can complicate the wound situation and delay the healing process [141]. The topical application of the antimicrobial component allows the appropriate dose over an extended time to be administered directly to the area of infection. Also, topical application minimizes the toxic effects of the drug [142]. Terbinafine is a synthetic allylamine antifungal agent that exerts its antibacterial activity by inhibiting the biosynthesis of ergosterol. It is effective in the treatment of various fungal infections, including oral candida. Szabo et al. developed a bioadhesive mucosal delivery system of PVA and CS loaded with the antifungal agent terbinafine. Terbinafine hydrochloride itself is hydrophobic and poorly soluble. Hydrophilic polymer-based nanofibers enable rapid and complete dissolution of drugs, which can overcome the dissolution problem of terbinafine. Furthermore, the oral absorption pattern of active substances such as terbinafine is highly dependent on the dosing conditions. Comparing several different administration conditions, it was found to be most beneficial when the expectorant mode of administration was maintained. Absorption from the oral mucosa was limited during the expectorant mode of administration, and gastrointestinal side effects were avoided. Mucosal administration of nanofibers minimizes the absorption of terbinafine in the oral cavity and improves drug utilization. This means that the local release of antifungal drugs in the oral mucosa is possible with nanofiber films [111].

Candida albicans is a common pathogenic organism in oral diseases and shows significant resistance to common antifungal drugs. Surprisingly, fatty acids naturally secreted by the microorganism have been shown to have antifungal properties. Therefore, Clitherow et al. attempted to use saturated fatty acids as antifungal agents to overcome resistance. Medium-chain saturated fatty acids were found to be the most promising alternatives to current antifungal drugs. PCL/PVP RS100 oral patches were prepared by high-voltage electrospinning, and nonanoic and dodecanoic acid was successfully loaded into the nanofibers. PVP absorbed water and swelled to achieve mucosal adhesion. Nanofibrous films containing dodecanoic acid showed the strongest fungal activities towards Candida Albicans (SC5314 and CAR17). This provides a promising new approach for oral ulcer treatment [125]. In conclusion, the antibacterial component is an important part of promoting the healing of oral ulcers.

#### 4.3.3. Natural Plant Extracts

The inflammatory response is detrimental to ulcer healing. Plant extracts interact with each other through different biochemical pathways to achieve anti-inflammatory, antioxidant, antifungal, and analgesic effects. Natural plant extracts are a rich treasure trove [143]. Countless researchers have worked to reveal the relationship and secrets of natural resources such as plants, animals, and even minerals to human health. Inspired by the long history of Chinese medicine, Chinese scientists Youyou Tu and others have creatively extracted the active ingredient of Artemisia annua, which yielded highly specific antimalarial effects. Natural plant extracts have great medical potential. The constituents of Ziziphus jujuba stem bark include terpenoids, quercetin, and tannins. Among them, tannic acid is suitable for treating oral inflammatory diseases [144]. Hosseinzadeh et al. innovatively prepared PAN nanofiber films loaded with Ziziphus jujuba extract by electrospinning. The nanofibrous films released 80% of the Ziziphus jujuba extract within 60 min and were shown to have anti-inflammatory effects. The nanofiber film also has good adhesion and mechanical properties, requiring about 4 N/m^2^ to separate from the mucosa. Anti-inflammatory and antibacterial effects were demonstrated when applied to periodontal disease treatment [4]. Chewable tablets containing Acacia catechu extract have a therapeutic effect on mouth ulcers [145]. Similarly, the ingredient is also expected to promote the healing of oral ulcers. GM contains a variety of flavonoids, benzophenones, and tannins with medicinal properties [146]. As one of the traditional medicinal herbs, GM has good antioxidant activity and antibacterial activity. Samprasit et al. prepared mucoadhesive electrospun CS and CS-SH nanofiber films using the synergistic antibacterial effect of GM extract and CS. The study demonstrated that this nanofiber film has good adhesion and can rapidly release active substances, thus reducing bacteria in the mouth [110].

#### 4.3.4. Corticosteroids

Corticosteroids can reduce the inflammatory response. Topical administration of corticosteroids has been proven to be effective in treating mouth diseases. In addition, topical administration not only allows for sustained release but also reduces side effects [147]. Triamcinolone acetonide is an anabolic corticosteroid commonly used to treat various skin conditions or to relieve oral ulcers [148]. The high-humidity environment of the oral cavity places high demands on the adhesion properties. Therefore, EC and alcohol soluble proteins have been investigated for the preparation of nanofibers. Alipour et al. added tretinoin to alcohol-soluble protein/EC nanofiber films for application in the treatment of oral ulcers. The results demonstrated that the addition of tretinoin to the nanofibrous films had anti-inflammatory effects and significantly reduced the levels of proinflammatory cytokines. Meanwhile, the addition of tretinoin improved the mechanical strength and thermal stability of the nanofibrous mat. The interaction of trimethoprim with alcohol-soluble proteins and EC increases the porosity of the nanofibrous mat [139]. This inspiration can reduce the pain and inflammation of oral ulcers by corticosteroids.

#### 4.3.5. Components That Promote Cell Proliferation

The entire cycle of oral ulcers includes primary injury response, signal amplification, ulcer formation, and healing. The healing process consists of epithelial cell migration, proliferation, and differentiation into a newly constructed extracellular matrix. To shorten the duration of oral ulcers and improve patient compliance, prohealing components can be added to the nanofibers to accelerate cell proliferation.

**Figure 12:**
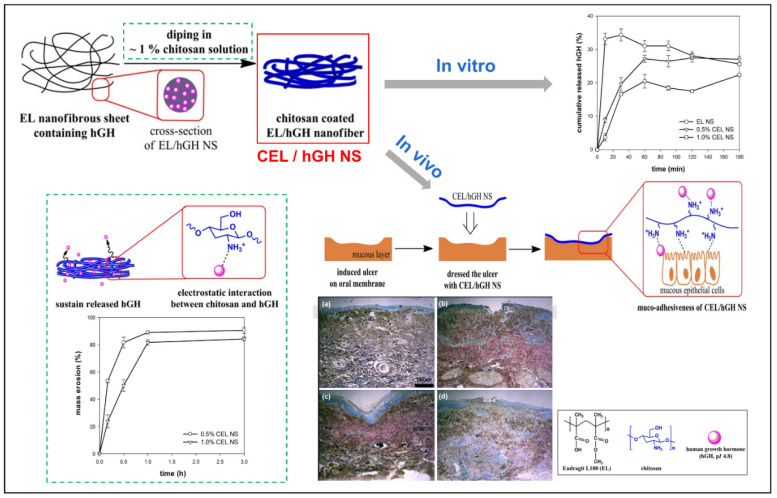
Schematic diagram of CEL/hGH-NS applied to the treatment of oral ulcers, reprint permission from Ref. [32]. Copyright 2016, John Wiley and Sons Inc.

hGH is a protein secreted by the pituitary cells and a peptide hormone that significantly increases the proliferation of human dermal fibroblasts. cEL/hGH-NS, a bilayer nanofiber patch composed of Eudragit and CS, was electrospun by Choi et al. (Figure 12). CS has excellent bioadhesive properties. hGH was first added in Eudragit and was co-electrospun into nanofiber films. Then, the films were impregnated and coated with CS to form CEL/hGH-NS. The CS layer causes a slow release of hGH from CEL/hGH-NS. Positively charged CS is electrostatically attracted by negatively charged hGH. The released hGH is captured in large amounts on the CS layer and remains on the oral mucosa for a long time. In addition, the CS layer significantly weakens the mass erosion of CEL/hGH-NS, delaying it to 1 h. In contrast, the sheet without CS coating melts immediately under physiological conditions. Tissue sections from an in vivo animal model of beagle show that 0.5% CEL/hGH NS grows the thickest regenerative epithelium [32]. Besides, niosomes gel encapsulating melatonin can treat oral mucositis triggered by oncology drugs (5-fluorouracil) [149].

A study has found that glutamine levels are reduced in patients with oral ulcers [150]. Glutamine is a nonessential amino acid in the body that provides energy for cell proliferation. As a precursor for the synthesis of mucin, glutamine promotes ulcer healing. The topical or systemic use of glutamine is beneficial in reducing the severity of oral mucositis [151]. Tort and Acarturk electrospun PEO-SA nanofibers loaded with glutamine for the treatment of cancer-induced oral ulcers. The nanofibers showed good mucosal adhesion, mechanical properties, stability, and release of more than 85% of the drug within 4 h [3]. Therefore, amino acids are also a promising option for oral ulcer treatment protocols.

## 5. Other Techniques in the Treatment of Oral Ulcers

### 5.1. Solvent Casting

**Figure 13:**
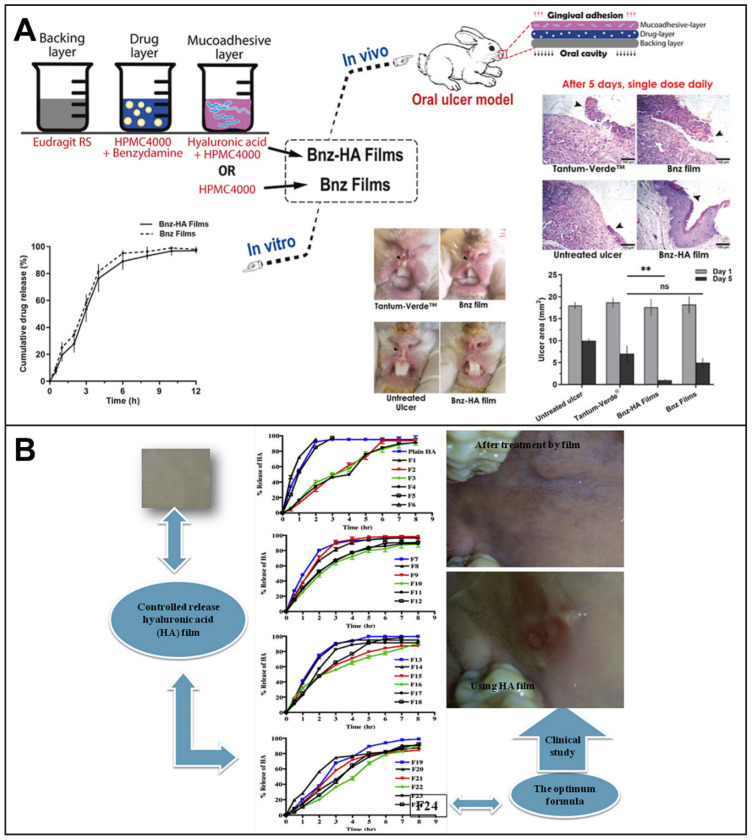
Preparation of oral adhesive films by solvent casting. (**A**) Bnz-HA films, reprint permission from Ref. [152]. Copyright 2021, Elsevier; (**B**) Oral mucosa adhesion film containing HA, reprint permission from Ref. [108]. Copyright 2020, Elsevier. **, *p* > 0.05.

Solvent casting is a classical technique for the preparation of membranes. This technique has long been investigated for the preparation of adhesive film patches in the oral cavity. Inspired by benzydamine mouthwash, EI-Salamouni et al. prepared a three-layer film (Bnz-HA) loaded with the drug benzydamine using a solvent-casting technique. The polymer of this three-layer film was used HA, and HPMC for the rapid topical treatment of oral ulcers (Figure 13A). In this case, the adhesive layer, intermediate drug layer, and a backing layer cooperated to achieve excellent adhesion and unidirectional drug release. The results demonstrated that the Bnz-HA film controlled the release of benzydamine and promoted the healing of oral ulcers with better results than mouthwash [152]. The bilayered mucoadhesive buccal patch prepared by the solution-casting method has been shown to allow systemic administration of domperidone. HPMC and PVP were used for the primary layer polymers, and Eudragit and PEO were used for the secondary layer polymers. This oral patch has suitable adhesion and permeability, and in vitro drug release also exhibits good release behavior [153]. The environment in the oral cavity is anaerobic and therefore most of the bacteria in the oral cavity are anaerobic or partly anaerobic. Metronidazole (MET) has been successfully used for over 45 years to treat anaerobic infection [154]. MET is often added to nanofibers for wound-dressing application [155]. OD is a newer generation of MET with a better antibacterial effect. DEX is a synthetic corticosteroid, which is inexpensive and has pharmacological effects, such as anti-inflammatory, antiendotoxic, immunosuppressive, antishock, and enhancing stress response. Zhang et al. studied the combined use of two functional components, OD and DEX, to achieve antibacterial and anti-inflammatory effects. In this study, a bilayer oral mucosal adhesion film with good mucosal adhesion properties was prepared using the solution-casting method. The backing layer uses the hydrophobic polymer EC, which is used to control the direction of drug release. The adhesive layer uses polymers HPMC, PVA, and CS, which are used to ensure adhesion of the membrane and to extend the drug-delivery time. The membrane thickness was 0.427 ± 0.015 mm, weight was 55.89 ± 0.79 mg, and surface pH was 6.34 ± 0.01. The drug content was 2.959 ± 0.106 mg/cm^2^, and more than 95% of the drug was released within 4 h. The fastest reduction in ulcer area was observed in a rabbit oral ulcer model with a double-layer film containing both drugs, with basic healing within 12 days. This composite film has promise as a topical drug delivery device for the treatment of oral ulcers [138]. HA not only activates the inflammatory response but also promotes cellular and angiogenesis. Huang et al. prepared an oral mucosal adherent film by casting a polymer solution containing HA into a glass culture dish. The freezing and thawing operations were then repeated to reduce drug release. Stable and suitable properties including homogeneity, folding tolerance, adhesion properties, drug-release rate, and mechanical properties were obtained by continuous optimization of parameters. Clinical studies have shown that the film resists the effects of saliva and oral activity and has a healing effect on oral ulcers. Over 99% reduction in ulcer area in volunteers after 7 days (Figure 13B) [108]. HA, HPMC, PVP, PEO, Eudragit, Gel, CMC-Na, PVA, and CS are all polymers that can be successfully electrospun into nanofibers. Moreover, these polymers contain a lot of functional groups that can form ionic or hydrogen bonds with biologically active substances. Therefore, it can adhere to the oral mucosal.

High-voltage electrospinning technology can be a very simple alternative to the solvent-casting technique. Nanofiber films prepared by electrospinning have better drug-delivery capability than films prepared by solution casting using the same polymer. The superiority of nanofibers in drug delivery for the treatment of oral ulcers is reflected in many aspects. First, the high specific surface area and porosity improve the adhesion and permeability of nanofiber films. Secondly, bioactive ingredients loaded on nanofibers can protect their activity. Third, nanofiber films have thinner dimensions and better flexibility than membranes. Fourth, the dissolution rate of insoluble drugs can be improved by generating amorphous solid dispersions in nanofibers. In conclusion, high-voltage electrospinning can greatly improve the performance of such oral adherent nanofiber films.

### 5.2. Cross-Linking

**Figure 14:**
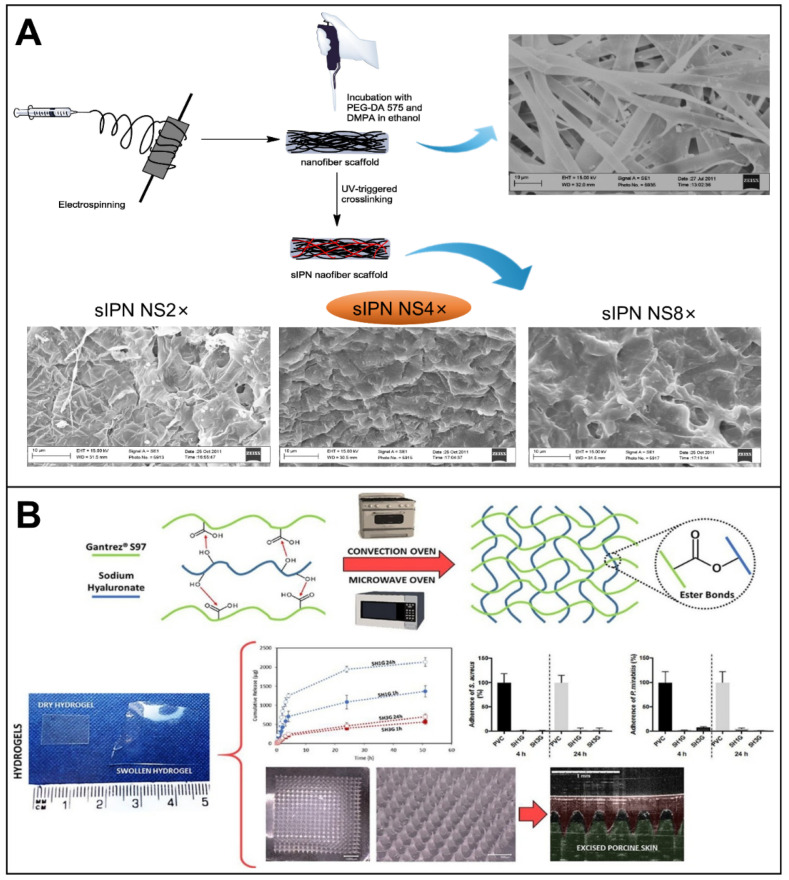
Cross-linking of nanofibers and hydrogels in the treatment of oral ulcers. (**A**) Photo-crosslinking of nanofibers, reprint permission from Ref. [121]. Copyright 2013, Elsevier; (**B**) Hydrogel [159].

Nanofibers prepared by high-voltage electrospinning can be cross-linked by physical or chemical means to improve the properties of the nanofibers. To improve the mechanical properties of Gel nanofibers can be photo-cross-linked. Gel nanofibers were first prepared by high-voltage electrospinning, then modified with photoreactive PEG-DA575 as a cross-linking factor to form semi-interpenetrating Gel nanofiber scaffolds (sIPN NSs). This nanofiber scaffold preserved the nanofiber morphology and improved the structural stability (Figure 14A). Among different cross-linker concentrations, sIPN NS4× exhibited the best mucin uptake and slow drug-release rate. In conclusion, the mechanical properties of sIPN NSs were greatly improved by photo-cross-linking, allowing them to adhere stably to the oral mucosa for slow drug release [121]. The nanofibers prepared by high-voltage electrospinning can be cross-linked to obtain good mechanical and drug-delivery properties, etc.

In addition to nanofibers prepared by high-voltage electrospinning, hydrogels are one of the promising bioadhesive drug-delivery forms. Hydrogels are prepared by chemical cross-linking or physical cross-linking. The polymer dissolves in water to an equilibrium state and its cross-linked network structure resists further dissolution, thus maintaining the hydrogel state. Hydrogels not only absorb and retain exudate but also promote fibroblast proliferation and keratin formation [156]. Singh and Dhiman synthesized a hydrogel using a chemically induced cross-linking method. Carbopol provides mucosal adhesion, polyvinylimidazole provides antimicrobial properties, and gum acacia provides antioxidant properties. This hydrogel possesses mucosal adhesion, antimicrobial, and antioxidant properties [124]. The following year, Singh et al. prepared antibiotic-loaded TG-PVA-PVP hydrogel dressings by radiation-induced cross-linking in an aqueous reaction system. The three-dimensional interpenetrating polymer network made the films flexible and elastic. The films achieve mucosal adhesion, absorption of wound exudate, and slow release of drugs. Furthermore, the film exerts a synergistic effect of antibacterial and antioxidant [117]. The anionic TG and cationic CS form a hydrogel. It combines the hydrogel properties of natural substances and electrostatic interactions to achieve adhesion in the oral mucosa [113]. Gel and CS cross-linked hydrogels can maintain a stable extracellular matrix-like structure for a long time [157]. A natural polysaccharide-based hydrogel combining CS and agarose holds promise for biomimetic remineralization of human tooth enamel [158]. Larraneta et al. first prepared HA-based hydrogels using an aqueous mixture of the polymer HA and the chemical cross-linker Gantrez S97. Then, the reaction was accelerated by the solid-phase synthesis in an oven and microwave radiation in a microwave oven (Figure 14B). Compared with other cross-linking methods, this method is safe, environmentally friendly and fast. The preparation process does not require the use of any organic reagents or substances. The results demonstrate that the cross-linked HA-based hydrogels have excellent antibacterial activity and are capable of sustained slow drug release. In addition, the microneedle array of the hydrogel can be used for transdermal drug delivery [159]. In conclusion, mucoadhesive drug delivery based on soft and moist hydrogels has great potential in oral mucosa drug delivery. This provides a promising new idea for the treatment of oral ulcers. Numerous natural substances that are difficult to electrospin can be prepared directly as hydrogels and then combined with nanofiber films. The advantages of different techniques are combined to achieve long-term adhesion and controlled drug release, as well as to reduce costs.

### 5.3. Lyophilization Method

**Figure 15:**
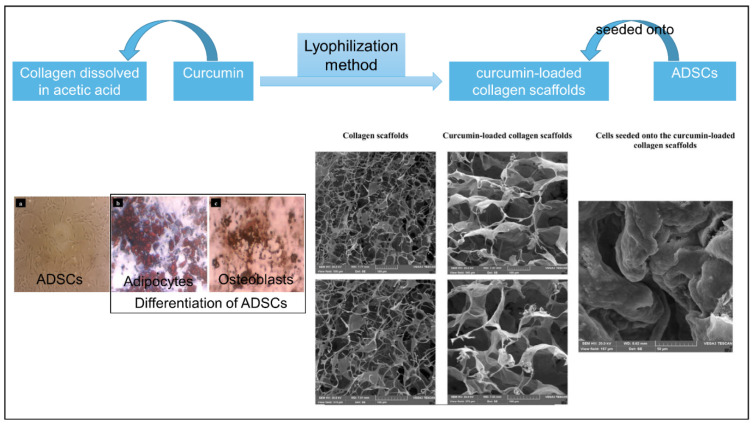
Collagen scaffold prepared by lyophilization method for the treatment of oral ulcers [31].

Stem cells can promote tissue repair through their ability to secrete various cytokines, chemokines, and growth factors. ADSCs represent progenitor and precursor cells for immature or undifferentiated cells of body tissues in a promising way [160]. ADSCs have been shown to have the potential for the treatment of oral ulcers [161]. CUR, the active component of turmeric, has shown healing-promoting effects when applied topically in animal models of mouth ulcers [162]. Although CUR is poorly water soluble, the core–shell nanofibers are used to achieve its sustained release [163]. Mardani et al. first obtained a collagen scaffold loaded with CUR directly by the lyophilization method (Figure 15). The scaffold allows not only adequate transport of gases and nutrients but also cell viability, propagation, polarization, migration, and protein synthesis. Then, the scaffolds were inoculated with ADSCs. It was demonstrated that ADSCs can differentiate into adipocytes and osteoblasts. Finally, the CUR–collagen scaffold inoculated with ADSCs was shown to significantly reduce the inflammatory response of mouth ulcers in a rat model [31].

## 6. Current Challenges

The high-voltage electrospinning technique has unique advantages in creating nanofibers [164,165,166], which have shown great potential in the treatment of oral ulcers and in providing all kinds of drug controlled-release profiles [167,168,169,170]. However, the application is still in the research phase and many challenges remain for further development. (1) The exact etiology of oral ulcers cannot be determined and there are obviously individual differences between patients. Therefore, a uniform composition cannot be chosen to cope with all patients. (2) The electrospun solution of drug-loaded nanofibers needs to be dissolved in some organic solvents. For example, CS should be dissolved in acidic solvents. Most of the solvent evaporates during the electrospinning process, but a complete absence of residues cannot be guaranteed. The impact of residue solvents on biomedical applications needs to be further explored. (3) The current in vivo evaluation of this type of study is incomplete. Most in vivo models are based on rats or rabbits, but the real environment of the human oral cavity is far more complex. (4) Oral ulcers are a common disease, and responding to the actual demand for high-volume preparation remains a challenge. Although the innovation of DRSNS has initially achieved the preparation of nanofibers in large quantities, the quality and drug distribution of nanofibers cannot be uniform. To resolve these issues, more efforts on the polymeric and lipid excipients and the combinations of nano methods with traditional pharmaceutical techniques are desired [171,172,173,174,175].

## 7. Conclusions and Outlook

So far, high-voltage electrospinning has continued to make breakthroughs in the preparation of nanofibers. The potential of nanofibers in the biomedical field is still being explored. On the one hand, high-voltage electrospinning technology has unique advantages. It is simple to operate and enables microscopic nanostructure design and drug loading in nanofibers in a single step. Electrospinning technology has many possibilities in terms of polymer selection and structural design. On the other hand, the nanoscale fiber membrane has unique physical/chemical properties. Nanofiber films have a similar structure to ECM, which makes them porous, permeable, and aids in cell proliferation. The special nanofiber structure can lead to functional improvements, including improved solubility of insoluble drugs, controlled drug-release rate, and targeted drug delivery. Nanofiber films offer advantages in drug-delivery systems and have great potential for specific applications. In addition, as an emerging technology, high-voltage electrospinning has made significant breakthroughs in terms of energy efficiency and mass production.

High-voltage electrospinning has significant potential for the preparation of bioadhesive nanofiber films. However, studies applying nanofibrous membranes to the treatment of mouth ulcers are relatively few. The special environment of the oral mucosa determines that adhesive nanofiber films are most suitable for the treatment of mouth ulcers. The selection of the appropriate polymer allows the nanofibers to rapidly absorb water and swell and adhere to the wet mucosal surface. Nanofibrous films can be applied to mucosal surfaces of any shape and size. Topical administration of nanofibers increases the duration of drug action and avoids the first-pass effect and gastrointestinal reactions of some drugs. Current treatments for oral ulcers fall into two main categories: topical and systemic treatments. The treatment for common oral ulcers is a topical treatment, which is the most effective way to improve the symptoms of ulcers. It is the most effective way to improve the symptoms of oral ulcers. Topical administration applies the drug directly to the damaged part of the mucosa. Topical administration reduces inflammation, relieves pain, prevents secondary infection, and promotes ulcer healing. For frequent recurrence or long-term unhealed oral ulcers, systemic treatment is used to improve and regulate immune function. The mucoadhesive film allows local administration and systemic treatment through the oral mucosa, making it ideal for the treatment of oral ulcers. In conclusion, it is necessary to study the application of nanofiber films for the treatment of oral ulcers.

This review analyzes the properties of these nanofibrous films in terms of both polymer and active pharmaceutical ingredients. The therapeutic effects of these nanofiber films on oral ulcers in animal experiments or clinical trials are also presented. The polymers that achieve oral mucosal adhesion can be natural substances or synthetic polymers or mixtures of them. The active medicinal ingredients include analgesic and anti-inflammatory NSAIDs, antimicrobial agents, natural plant extracts, corticosteroids, etc. Suitable components and polymers can be selected from the summary, or new components can be developed to be added for electrospinning preparation. In addition, the materials and compositions used for such methods as solvent casting and cross-linking and lyophilization are of great reference value for electrospinning. Based on this, more combinations of polymers and loaded components of nanofibers can be inspired. These new combinations for the treatment of oral ulcers are expected to achieve better therapeutic effects. This not only opens up new possibilities for pharmaceutical nanofibers but also offers new hope for the cure of oral ulcers. In addition, this bioadhesive, biocompatible, biodegradable, antibacterial, analgesic, anti-inflammatory, and healing-promoting nanofibrous membrane has potential for other applications. The application potential includes topical delivery of drugs for breaks or ulcers in the gastrointestinal tract, urethra, vagina, etc., although there are still many challenges to be solved for the application of nanofibers prepared by electrospinning in oral ulcers. However, electrospinning technology has a broad future in the preparation of adherent patches for topical drug delivery and for expanding the applications of biomolecules [176,177,178,179].

## Figures and Tables

**Table 1:** Classification of recurrent oral ulcers.

Type	Symptoms	Pattern	Number	Distribution	Course
Mild oral ulcers	Tenderness with burning sensation	Clear borders	1–5 pcs	Mucosa	7–10 days
		Central slight depression	3–5 mm in diameter		
		Pale-yellow pseudomembrane			
Severe oral ulcers	Severe pain	Margins slightly elevated	1 or several	Soft palate pharynx	More than 1 month
		Deep central depression	More than 1 cm in diameter	Medial lip	
Herpes- type oral ulcers	Most painful	Reddening of blood	More than 10 mm	Mucosa	10–14 days
			Approximately 2 mm in diameter		

## Data Availability

Not applicable.

## Abbreviations

A/S	alkannin and shikonin
AAP	acetaminophen
ADSCs	adipose tissue-derived stem cells
CA	cellulose acetate
Car	carvedilol
CMC-Na	sodium carboxymethylcellulose
CMCS	carboxymethyl chitosan
CS	Chitosan
CS-SH	thiolated chitosan
CUR	curcumin
DRSNS	double-ring slit needleless spinneret
DS	diclofenac sodium
EC	ethyl cellulose
EL	Eudragit L
ES	Eudragit S
FDOFs	fast-dissolving oral films
Gel	gelatin
GM	*Garcinia mangostana*
HA	hyaluronic acid
hGH	human growth hormone
HPMC	hydroxypropyl methylcellulose
IBU	ibuprofen
KET	ketoprofen
KSNO	S-nitrosated keratin
MET	metronidazole
MOF	metal–organic framework
NO	nitric oxide
NSAIDs	nonsteroidal anti-inflammatory drugs
OD	ornidazole
PAN	polyacrylonitrile
PC	phospholipid
PCL	polycaprolactone
PEG	polyethylene glycol
PEO	polyethylene oxide
PGA	poly(α-L-glutamic acid)
PLA	polylactic acid
PLGA	polyglycolic acid
PP	polypropylene
PU	polyurethane
PVA	polyvinyl alcohol
PVP	polyvinylpyrrolidone
SA	sodium alginate
TG	tragacanth gum
WHO	World Health Organization
